# A case series of hyperthermia crisis in cough mixture ingestion: Why early cooling save lives

**DOI:** 10.1016/j.toxrep.2025.102168

**Published:** 2025-11-19

**Authors:** Hon Chun, Tin Yat Anthony Chow

**Affiliations:** a Department of Accident & Emergency, Pamela Youde Nethersole Eastern Hospital, Hospital Authority, Hong Kong Special Administrative Region of China; b Department of Clinical Toxicology, United Christian Hospital, Hospital Authority, Hong Kong Special Administrative Region of China; c Hong Kong Poison Control Centre, Hospital Authority, Hong Kong Special Administrative Region of China

## Abstract

**Objective:**

Hyperthermia (core body temperature >40°C) associated with cough mixture ingestion has been reported in case studies. However, clinical data and consensus on treatment remain limited. This study aimed to identify associated factors, characterize clinical manifestations, and evaluate management approaches for hyperthermia following cough mixture ingestion.

**Methods:**

A territory-wide retrospective case series was conducted using data from the Hong Kong Poison Information Centre (HKPIC) between 1 January 2013 and 31 December 2023. Patients aged > 18 years with cough mixture ingestion, hyperthermia (>40°C), and urine toxicology results were included. Cases with abusive stimulant co-ingestion were excluded.

**Results:**

Of 434 recorded cough mixture exposures, 156 had urine toxicology results. After exclusions, 9 patients were included (100 % male; median age 37.1 ± 9.0 years). Mortality was 33.3 % (3/9). All cases occurred in daytime with ambient temperatures > 36°C. Promethazine and ephedrine were detected in all patients. Cooling methods were used in 55.6 % (5/9).

**Conclusion:**

Promethazine/ ephedrine containing cough mixtures and high ambient temperatures may synergistically contribute to hyperthermia. Early recognition and aggressive cooling are critical to reduce mortality. Such cough mixtures should be restricted from being sold as over-the-counter medications.

## 1 Introduction

Cough mixtures are widely used in Hong Kong, where they can be purchased without a prescription from pharmacies and are consumed for both therapeutic and abusive purposes. Typical ingredients in locally available cough mixtures include antihistamines (e.g., promethazine，diphenhydramine, chlorpheniramine), cough suppressants (e.g., codeine, dextromethorphan, hydrocodone), and nasal decongestants (e.g., ephedrine, pseudoephedrine, phenylpropanolamine) [1]. Clinical toxicities associated with cough mixture ingestion commonly involve drowsiness, behavioral changes, electrolyte disturbances, and other systemic effects related to opioid or anticholinergic toxidromes [2], [3], [4].

Hyperthermia—defined as a core body temperature exceeding 40°C [5], —is a rare situation following cough mixture ingestion [6]. Non-pyogenic hyperthermia can be caused by environmental factors (hot weather, high humidity) [7] and various medications, including psychotropics (antipsychotics, antidepressants and anxiolytics), anticholinergics, antihistamines, diuretics, cardiovascular agents, non-steroidal anti-inflammatory drugs and anticoagulants [8]. Among the cough mixture contents, antihistamines and nasal decongestants may be associated with hyperthermia. Besides, abusive stimulants (e.g., cocaine, amphetamine, methamphetamine), which are occasionally co-ingested by cough mixture abusers, are well-known causes of hyperthermia [1]. In some situations, heat stroke may co existent with drug induced hyperthermia [9]. No matter what scenario, rapid cooling remains the cornerstone of the treatment in non-pyogenic hyperthermia [7], [10].

The current experience of cough mixture related hyperthermia is limited to only one prior case report, documenting its occurrence in a patient with exertional heat stroke [6]. There is no case series to systematically discuss demographic, clinical information, and treatments for hyperthermia with cough mixture ingestion. We aim to describe a rare series of hyperthermia cases (body temperature >40°C) following cough mixture ingestion (exclude abusive stimulants co-ingestion), a phenomenon that has rarely been documented in literature.

## 2 Method

### 2.1 Study population

This territory-wide retrospective observational study included patients reported to the Hong Kong Poison Information Centre (HKPIC) for drug overdose between 1 January 2013 and 31 November 2024. HKPIC served as Hong Kong’s sole poison control center during the study period. Inclusion criteria required all of the following: age ≥ 18 years, documented cough mixture ingestion, availability of urine toxicology results (done by Liquid Chromatography with tandem mass spectrometry (LC-MS/MS) from the current admission, and a recorded body temperature ≥ 40°C. Exclusion criteria comprised co-ingestion of abusive stimulants, as confirmed by urine toxicology.

**Data Collection** Demographic and clinical data were extracted from medical records, including:

• Patient background: onset time, exertion prior to admission, outdoor exposure
• Characteristics: age, gender, comorbidities, histories of substance abuser
• Ingestion details: time, estimated dose, co-ingested substances
• Clinical manifestations: symptoms, body temperature recordings in the Emergency Department
• Treatment: use of cooling methods in documentation
• Outcomes: prognosis, complications (e.g., rhabdomyolysis, acute renal failure, etc)
• Laboratory results: urine immunoassay/toxicology reports

Temperature and humidity data were obtained from historical weather station data (Hong Kong International Airport) via *timeanddate.com*, and ambient temperatures were calculated according to reference 11. Glasgow Coma Scale (GCS) scores were assessed using standardized criteria [12]. Bedside urine drug screening was performed using ABON® immunoassay devices.

## 3 Definitions

• Acute renal failure: defined per RIFLE classification [13].
• Rhabdomyolysis: serum creatine kinase > 5 × the upper normal limit [14].
• Disseminated intravascular coagulation (DIC): diagnosed using ISTH criteria [15].
• Exertional: different physical exertion linked tasks such as occupational work, recreational sports and events, and organized competitive sports [16].
• Outdoor: people who typically located under the sun or under partially shaded conditions [17].

## 4 Analysis

Patient demographics and clinical data were extracted and entered in a preformatted Excel database. Descriptive statistics were used with continuous variables reported as means and standard deviations (SD). Categorical variables reported as percentages.

## 5 Results

During the study period, 434 cases of cough mixture exposure were recorded in the database. Of these, 156 patients had urine toxicology results available. After excluding cases involving stimulant co-ingestion and patients with peak body temperatures below 40°C, nine patients were included in the final analysis (Table 1). The mean age was 37.1 ± 9.0 years, and all participants were male. Two patients had no history of cough mixture abuse, with at least one confirmed to have ingested the medication for therapeutic purposes. All cases occurred between late June and early September, coinciding with Hong Kong’s hottest months. Ambient environmental temperatures at the time of presentation exceeded 36°C for all patients. Of the nine cases, 66.7 % (6/9) were discovered outdoors, and 33.3 % (3/9) had engaged in strenuous physical activity (e.g., construction work or heavy lifting) prior to symptom onset.

**Table 1:** Characteristics of 9 patients with cough mixture ingestion and hyperthermia.

Case	Age Gender	Time	Ambient Temperature	Outdoor/ Exertional	Ingestion time and amount	Cough mixture abuser	Symptoms in arrival	Arrival body temperature	Bedside Urine Immunoassay	Urine toxicology	Cooling methods	Other treatments	Outcome and complications
1	46 M	12 am, 22-Jul-2013	38.7	-/-	unknown	+	LOC	40.2	Morphine, methamphetamine	Promethazine, Codeine, Ephedrine, Phenylpropanolamine	Nil	Intubation	Died of pneumonia (7 days later); ARF, Rhabdomyolysis
2	25 M	9 am, 30-Jul-2013	36.6	-/+	unknown	+	LOC, seizure	40.4	Amphetamine, ketamine, cannabis, opiate, morphine	Promethazine, Codeine, Ephedrine, Cannabinoids metabolite	Cold saline infusion	Intubation	Survive
3	32 M	11 am, 27-Aug-2016	40.4	+ /+	120 ml, 1 h ago	+	LOC, shock	41.0	methamphetamine, morphine	Promethazine, Codeine, Ephedrine	Nil	Intubation	Survive, with ARF, Rhabdomyolysis
4	45 M	2 pm, 19-Jul-2020	40.7	+ /-	unknown	-	LOC, then arrest	42.0	Not done	Promethazine, Chlorpheniramine, Codeine, Dihydrocodeine, Morphine, Oxycodone, amoxicillin, Ephedrine, Phenylpropanolamine	Nil	Intubation, inotropes	Survive, with permanent brain damage, ARF, Rhabdomyolysis
5	27 M	3 pm, 6-Jul-2021	40.1	+ /+	10 ml, 3 h ago	-	LOC, seizure	41.0	methamphetamine, tri-cyclin antidepressants	Promethazine Ephedrine, Codeine	Cold saline infusion, bladder irrigation, ice pack, benzodiazepine	Intubation	Survive, with rhabdomyolysis
6	33 M	6 pm, 30-Jun-2023	38.9	+ /-	unknown	+	LOC	42.2	Not done	Promethazine Ephedrine, Codeine, Amoxicillin	Nil	Intubation, inotropes	Died of cerebral edema (12 days later); ARF, DIC, Rhabdomyolysis
7	43 M	2 pm, 26-Aug-2023	40.4	+ /-	60 ml, unknown time	+	LOC, seizure	43.3	methamphetamine, opioid	Promethazine, Brompheniramine, Carbinoxamine, Chlorpheniramine, Codeine, Dextromethorphan, Ephedrine, Hydrocodone, Morphine, Phenylpropanolamine, Zopiclone	Cold saline infusion, bladder irrigation	Intubation	Died of pneumonia (18 days later); ARF, Rhabdomyolysis
8	50 M	7 am, 23-Jul-2024	36.3	-/-	120 ml, 6 h ago	+	LOC	41.8	methamphetamine, morphine	Promethazine, Codeine, Ephedrine, Morphine,	Cold saline infusion, ice pack	Intubation	Survive, with rhabdomyolysis
9	33 M	4 pm, 5-Sep-2024	40.3	+ /-	240 ml, 2 h ago	+	LOC	42.0	Not done	Promethazine, Ephedrine	Cold saline infusion, ice pack	Nil	Survive, with Rhabdomyolysis

GCS: Glasgow coma scale, LOC: loss of consciousness, ARF: acute renal failure.

All patients were found coma by bystanders, with one in cardiac arrest upon paramedics’ arrival. Seizures occurred in 44.4 % (4/9) of cases. Core (rectal) temperatures were monitored in patients receiving cooling interventions, while tympanic measurements were used for others. The mean initial body temperature recorded in the Accident and Emergency Department (AED) was 41.5 ± 1.0°C. Cooling measures—including cold saline infusion, ice packs, and bladder irrigation—were implemented in five patients diagnosed with heat stroke, all of whom underwent core temperature monitoring. As shown in Fig. 2, body temperatures declined rapidly post-intervention, though one patient experienced rebound hyperthermia. Among the four patients not receiving cooling therapy, only one exhibited a downward temperature trend.

**Fig. 1:**
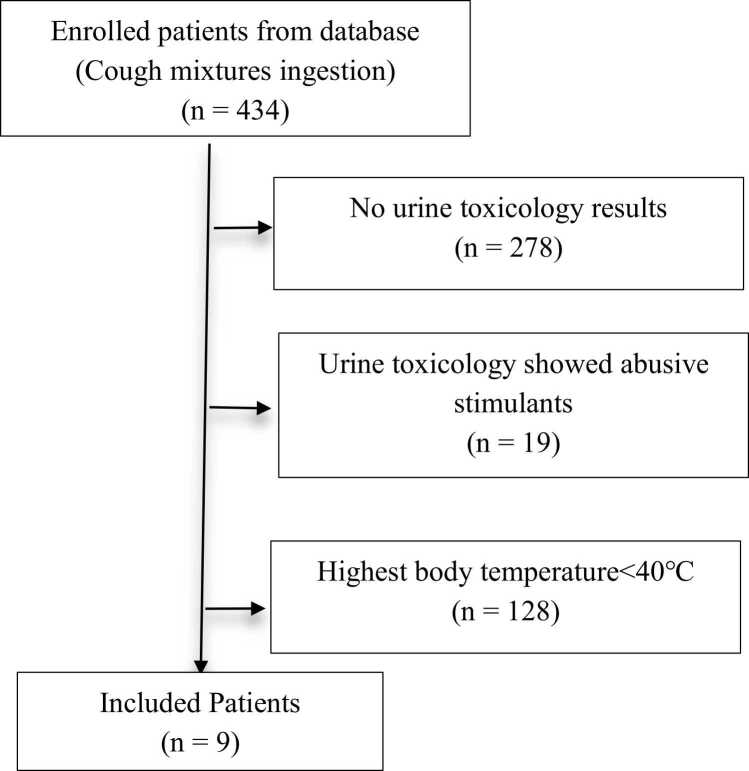
Flowchart of patients’ enrollment.

**Fig. 2:**
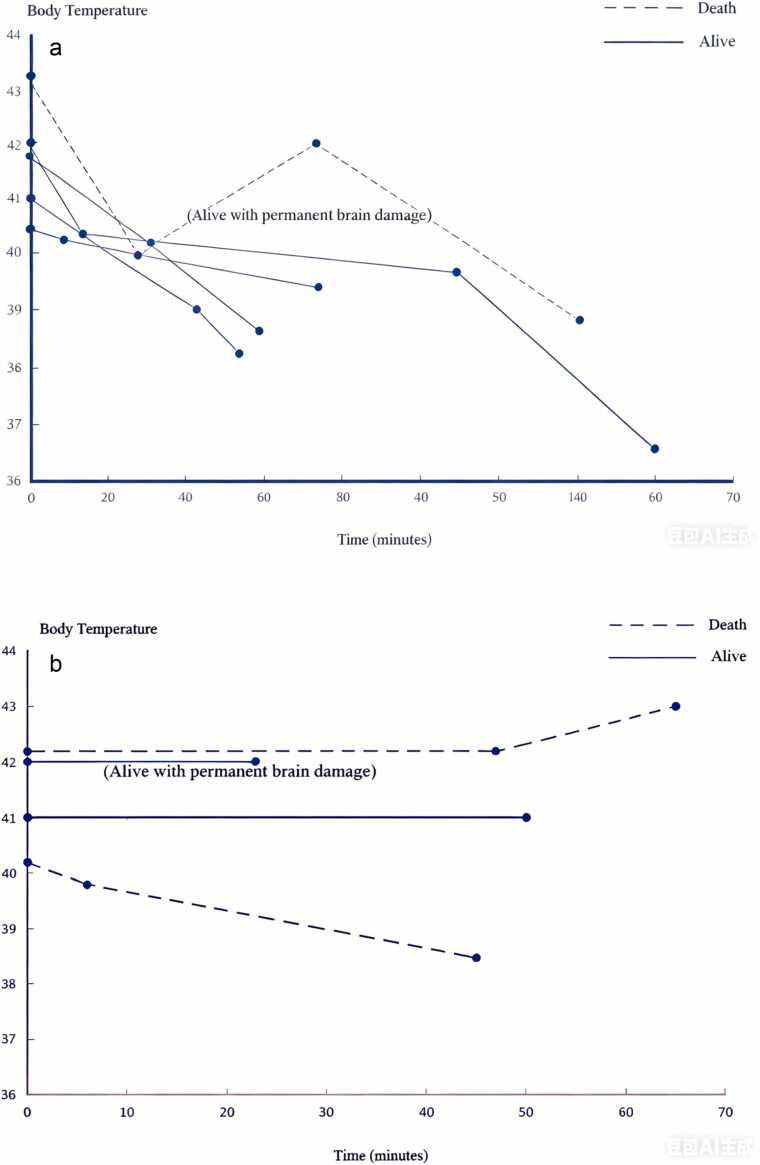
Body Temperature change in hyperthermia patients. 2a showed patients with cooling methods, and 2b showed patients without cooling.

5 out of nine patients received cooling, with all (5/5) of them with cold saline infusion, 60 % (3/5) with ice pack, 40 % (2/5) with bladder irrigation. Of the 5 patients receiving cooling, only one patient died (with a core temperature of 43.3°C and developed refractory hyperthermia despite interventions). In contrast, cooling was not documented in another 4 patients, with two of them died and one survived with permanent brain damage. Three patients died during hospitalization due to pneumonia or cerebral edema, with the longest survival time being 18 days after admission.

Complications included rhabdomyolysis (88.9 %, 8/9), acute renal failure (55.6 %, 5/9), and disseminated intravascular coagulation (11.1 %, 1/9). Most patients (88.9 %, 8/9) required ICU admission, with a median ICU stay of 3.8 ± 3.7 days. The median acute hospital stay was 9.7 ± 6.0 days, and the patient with permanent brain damage was transferred to a rehabilitation center for ongoing care.

Urine toxicology revealed promethazine and ephedrine in all cases (100 %, 9/9), with opioids detected in 88.9 % (8/9). By comparison, among all 156 patients with cough mixture exposure and toxicology data, promethazine was present in 76.2 % (119/156), ephedrine in 80.1 % (125/156), and opioids in 93.6 % (146/156) of patients. These findings align with the typical composition of cough mixtures, which frequently contain promethazine, ephedrine, and opioid analogues (e.g., codeine, morphine). Cannabinoid metabolites were identified in one patient.

## 6 Discussion

This case series highlights the critical association between cough mixture ingestion and life-threatening hyperthermia, particularly in the context of elevated ambient temperatures and specific pharmacological ingredients in cough mixture. Our findings suggest that promethazine, the most common antihistamine in cough mixtures locally, may play a central role in the development of hyperthermia, compounded by environmental heat stress. The high mortality rate (33.3 %) and frequent complications such as rhabdomyolysis, AKI, and DIC underscore the severity of this condition and the urgent need for early recognition and targeted management. Rapid and effective cooling is the most important treatment for hyperthermia associated with cough mixture ingestion.

The universal presence of promethazine in urine toxicology among our study aligns with its known anticholinergic properties, which impair thermoregulation by reducing sweating and cutaneous vasodilation [10], [18]. This mechanism likely synergizes with high ambient temperatures (>36°C), creating a "double-hit" effect that exacerbates hyperthermia risk. Similar interactions between anticholinergic drugs and heat stress have been reported in populations [19], [20], [21]. Outdoor environment and exertion also contributed to heat stress [17], which was also discovered in current study. Notably, all cases occurred during Hong Kong’s hottest months, emphasizing environmental heat as a critical precipitating factor, but this study uniquely identifies cough mixture abuse as another factor.

Ephedrine and codeine were also detected in most patients’ urine toxicology, reflecting their common inclusion in cough mixtures alongside promethazine. Although no prior research has directly linked hyperthermia to either ephedrine or codeine, their pharmacological properties suggest potential contributory roles. Ephedrine, a sympathomimetic agent, increases metabolic rate and induces vasoconstriction, which may elevate body temperature while reducing heat loss [22]. Codeine, through opioid-mediated respiratory depression and sedation, diminishes behavioral responses to heat stress and limits the body’s ability to dissipate excess heat [23]. Taken together, these two agents may act together with promethazine and environmental heat exposure to exacerbate the risk of hyperthermia.

In our study, all patients with cough mixture ingestion with hyperthermia were male, which is consistent with previous Hong Kong territory study [1]. Hyperthermia can be observed in both cough mixture normal user and abuser. Chronic usage may lead to cumulative anticholinergic effects [24], increasing susceptibility to hyperthermia. Furthermore, concurrent opioid exposure (which is also common cough depressant used in cough mixture) could exacerbate respiratory depression and reduce heat dissipation capacity during physical exertion [25], as observed in 33.3 % of patients engaged in rigorous activities.

Interestingly, 66.7 % patients had bedside urine immunoassay test for drugs, and all of them showed stimulants (amphetamine, methamphetamine) positive, which may be associated with false positive produced by ephedrine, a common content in cough mixture [26]. Nevertheless, no matter if cough mixture or stimulant associated hyperthermia, crucial treatments should be cooling [27], [28]. Despite hyperthermia, suboptimal utilization of cooling measures (44.4 % of cases) likely contributed to poor outcomes.

For treatments, recognizing the hyperthermia status is the 1st step. Cooling is crucial for cough mixture associated hyperthermia treatment. Mortality is higher in patients without cooling (50 %) compared to patients with active cooling (20 %), which signifies the importance of prompt cooling. This aligns with established guidelines for heat stroke and drug-induced hyperthermia, where rapid temperature reduction is paramount to prevent end-organ damage and mortality [29]. Notably, the single death in patients with cooling occurred in one patient with refractory hyperthermia (43.3°C), highlighting that while cooling improves survival, delays in initiation or extreme initial temperatures may limit efficacy. The observed rebound hyperthermia further emphasizes the need for continuous monitoring and sustained cooling until thermoregulatory stability is achieved. Despite the small cohort, these findings reinforce that cooling is not merely adjunctive but lifesaving, as delayed or absent intervention allows unchecked hyperthermia to drive complications such as rhabdomyolysis, acute kidney injury, and cerebral edema.

## 7 Limitations

This study has several limitations. First, the small sample size (n = 9) precludes definitive causal conclusions. Second, retrospective design limits data granularity, including exact doses ingested and temporal relationships between ingestion and hyperthermia onset. Third, the absence of a control group (e.g., cough mixture users without hyperthermia) hinders the identification of independent risk factors.

## 8 Conclusion

This study identifies promethazine/ ephedrine containing cough mixtures as potential catalysts for severe hyperthermia during environmental heat exposure. A paradigm shift toward recognizing this situation as both a toxicological and environmental emergency may improve outcomes in this vulnerable population. In view of medical safety concerns and the potential for abuse, the sale of cough mixtures containing promethazine and codeine should be restricted and not allowed as over-the-counter medications in the future.

## Ethics and informed consent statement

This study was approved by the Kowloon Central / Kowloon East Cluster Research Ethics Committee of Hospital Authority, Hong Kong (Ref No.: CIRB-2025–023–1). Informed consent was waived by the Committee due to the retrospective design and use of anonymized data.

## Funding

Nil

## CRediT authorship contribution statement

**Chow Tin Yat, Anthony:** Writing – review & editing, Supervision, Resources, Methodology, Conceptualization. **Chun Hon:** Writing – review & editing, Writing – original draft, Methodology, Formal analysis, Data curation, Conceptualization.

## Declaration of Competing Interest

The authors declare that they have no known competing financial interests or personal relationships that could have appeared to influence the work reported in this paper.

## Data Availability

Data will be made available on request.
